# Ash from Poultry Manure Incineration as a Substitute for Phosphorus Fertiliser

**DOI:** 10.3390/ma15093023

**Published:** 2022-04-21

**Authors:** Magdalena Cempa, Paweł Olszewski, Krzysztof Wierzchowski, Piotr Kucharski, Barbara Białecka

**Affiliations:** Central Mining Institute, 40-166 Katowice, Poland; polszewski@gig.eu (P.O.); kwierzchowski@gig.eu (K.W.); pkucharski@gig.eu (P.K.); bbialecka@gig.eu (B.B.)

**Keywords:** fertiliser, phosphorus, ash, poultry manure, anthropogenic land

## Abstract

The goal of the tests was to separate a phosphate concentrate from ash and to assess its fertiliser use efficiency in anthropogenic land. Ash obtained from poultry manure incineration is an interesting fertiliser, as it contains both of the necessary nutrients, i.e., phosphorus and potassium. The ash selected for the tests contained 15.73 wt% P_2_O_5_, and 6.75 wt% K_2_O. CaO also constituted the main component (44.79 wt%). Phosphorus in crystalline form was present as hydroxyapatite and carbonate apatite. The first stage, applied in order to separate a phosphate concentrate from ash, involved a number of physicochemical methods: (i) a method based on grain wettability differences; (ii) a method based on grain density differences; and (iii) methods based on size distribution differences. Wet sieving made it possible to separate a fraction with a P_2_O_5_ content of 24.56 wt%. The second stage, applied to assess fertiliser use efficiency, involved cassette tests as well as pot and field cultivation using as fertiliser, the obtained product as well as raw ash and commercial ones. Therefore, the conducted research allowed for the development of a methodology for the management of ash from the incineration of a poultry manure and their use as a substitute for phosphorus fertiliser. The tested material was applied in various doses. Using the obtained phosphate concentrate at a dose of 95 g/m^3^ resulted in a comparable yield as in the case of the commercial fertiliser at a dose recommended by the producer (75 g/m^3^). Unprocessed ash had to be used in larger amounts, i.e., 165 g/m^3^, to have a comparable yield as a commercial fertiliser.

## 1. Introduction

Poland is currently one of the leading producers of poultry meat and eggs in Europe [1]. In the years 2000–2020, there was a significant increase in chicken production in Poland, from 48.3 million to 182.5 million [2,3]. Assuming that the quantity of excrements produced by one bird is about 100 g/day [4], it is estimated that a total of 0.13 million tonnes of fresh chicken manure is generated weekly [5]. Such a great mass of waste constitutes a significant problem for the environment and requires appropriate action in both technical and legal directions. It should be stressed that phosphorus-rich waste, including poultry manure and ash, remaining after its incineration, should be treated as a raw material to be used in other branches of the economy, as per the idea of the circular economy promoted by the EU [6,7,8,9].

There are a number of options for the management of poultry manure or litter, and a few of them are of particular importance: using it as a fertiliser in agriculture, for recultivating soil poor in organic matter, for obtaining energy, or as a fodder addition for other animal groups, e.g., beef cattle or fish [10,11,12,13,14,15,16,17]. However, current applicable legislation [18] prohibits the unlimited use of this type of waste. The reason for this is primarily the resultant possibility of water contamination and eutrophication [19]. The use of poultry manure as a fertiliser can also generate greenhouse gas emissions due to the release of methane and ammonia [14].

In recent years, manure incineration has been adopted as an alternative for its agricultural use, with the purpose of producing energy and thereby reducing the waste mass by about 80%. The incineration process simultaneously eliminates all biological hazards as well as inconveniences related to odours [20,21,22,23,24,25].

The content of elements such as phosphorus and potassium in ash originating from poultry manure or litter incineration can vary due to the type of poultry production, type and quantity of feed, type of bedding, number of birds in a flock and the incineration process conditions [26,27,28]. On average, the ash contains 2%–10% phosphorus (P), 12%–32% calcium (Ca) and 6%–15% potassium (K), which contributes to its common acknowledgement as a significant source of nutrients for crops [29,30,31].

Composition analyses of ash obtained from poultry manure incineration conducted by [5,11,32] led to a number of significant conclusions: (i) phosphorus was detected only in coarse-grained fractions, hence the necessity of dividing the ash into fractions and separating the phosphate concentrate; (ii) the phosphorus-containing crystalline component was always hydroxyapatite as well as whitlockite; and (iii) the source of the bioavailable phosphorus form contained in poultry manure ash is the amorphous phase. The ash also contains heavy metals originating from poultry bedding, primarily copper (Cu) and zinc (Zn), which may constitute a significant obstacle for its direct use as a fertiliser, depending on local legislation [18,33,34].

The directions for using poultry manure are numerous, but the solutions that bring economic benefits with simultaneous care for the environment will continue to gain prominence each year. Such solutions include incinerating manure and using the obtained ash to produce new products of great value, including for the purposes of soil fertilisation. The agricultural use of ash from poultry manure or litter incineration, with minimum processing, can make it a cost-effective and attractive substitute for commercial fertilisers. The ash is free of pathogens and toxic organic substances such as pharmaceuticals, whereas the ratio of heavy metals to phosphorus in the ash is the same as in the poultry manure, while the ash is more concentrated [30]. However, recycling ash and thereby closing the P and K cycle is subject to regulations concerning the use of waste materials as fertilisers [35,36,37].

The goal of the tests was to separate a phosphate concentrate from ash and to assess its fertiliser use efficiency on anthropogenic land. The first stage, applied in order to separate a phosphate concentrate from ash, involved a number of physicochemical methods: (i) a method based on grain wettability differences; (ii) a method based on grain density differences; and (iii) methods based on size differences. The second stage, applied to assess fertiliser use efficiency, involved cassette tests as well as pot and field cultivation using, as fertiliser, the obtained product as well as raw ash and commercial ones. The fertilisers were applied in various doses to select the optimal one.

## 2. Materials and Methods

### 2.1. Materials

The ash from poultry manure incineration (PMA) was collected from an industrial plant with the following working parameters:

- incineration technology: circulating fluidised bed combustion,
- fuel: laying hens’ manure,
- fluidised bed composition: CaCO_3_ sand and recirculated ash [38,39].

A sample (about 50 kg) was collected from a cyclone and electrostatic precipitator, brought to an air-dry state and averaged for tests.

The anthropogenic land soil (SW) was collected from ten different points at the base of the “Waleska” coal waste dump in Łaziska Górne (Poland), to an amount of about 200 kg. The test sample was brought to an air-dry state, screened through a 2 mm sieve and averaged for testing.

The tests also utilised: citric acid p.a. (Chempur), sulphuric acid p.a. (Chempur), hydrofluoric acid p.a., nitric acid p.a. (Chempur), tribromomethane p. (WarChem), tetrachloroethene p. (WarChem), xylene p. (WarChem) as well as a mixture of grass (MG), reference soil (SR), flotation reagent (FR-A, FR-B, FR-C, FR-D) and commercial fertilisers (CF-S, CF-A) (Table 1).

### 2.2. Characteristics of Ash

#### 2.2.1. Analytical Procedure

The following was determined for the ash sample:

- Total carbon, total hydrogen and total sulphur content via high-temperature incineration with IR detection using a CHS900 elemental analyser (ELTRA, Haan, Germany); the thermal sample decomposition was carried out at a temperature of 1450 °C in an oxygen atmosphere;
- Ash content by weight; sample incineration was carried out at the temperature of 815 °C in a laboratory furnace;
- Total nitrogen content by Kjehdahl’s method with Deward’s alloy addition;
- Main chemical component and trace element content by wavelength dispersive X-ray fluorescence spectroscopy (WDXRF) using a ZSX PRIMUS II analyser (Rigaku, Tokyo, Japan) equipped with a 4 kW X-ray Rh tube; the samples were prepared by borate fusion (1 g sample: 9 g flux), the beads were obtained by melting the resulting mixture at a temperature of 1050 °C;
- Phosphorus and calcium content by inductively coupled plasma optical emission spectroscopy (ICP-OES) using an Elmer Optima 5300 analyser (PerkinElmer, Waltham, MA, USA); the analysis was performed after mineralising the material in a mixture of acids prepared according to the standard [43];
- Bioavailable phosphorus content via extraction in citric acid; the sample was mixed with 2% citric acid solution at a ratio of 1 g: 100 mL with an accuracy of ±0.001 g and 0.05 mL, respectively; the sample was centrifuged on a rotary mixer at a speed of 80 rpm for a period of 30 min.; the sample was then centrifuged for 10 min and filtered using MCE membrane filters (pore size 0.45 μm; Whatman, Maidstone, UK) to obtain a clear solution; total phosphorus content was determined by the Phosphate–Phosphomolybdenum blue method using a Nanocolor 500D photometer (Macherey-Nagel, Düren, Germany) [44];
- Mineral composition by powder X-ray diffraction (XRD) in Bragg-Brentano geometry using a D8 DISCOVER diffractometer (Bruker, Billerica, MA, USA) with a CuKα lamp, Ni filter and a LYNXEYE_XE detector working under the following conditions: voltage–40 kV, 2theta angle step size–0.01°, time–1 s by step, 2theta angle range 4–69°; sample rotation–10°/min; the composition was calculated on the basis of patterns licensed in PDF-4+ 2021 RDB ICDD (International Centre for Diffraction Data) and databases: ICSD (Inorganic Crystal Structure Database) and NIST (National Institute of Standard and Technology); the following programs were used for registration and diagnostics: DIFFRAC v.4.2 and TOPAS v.4.2. Bruker AXS; the quantitative phase composition was determined by the Rietveld method;
- Grain surface morphology analysis and chemical composition in micro-areas by scanning electron microscopy (SEM) and X-ray energy dispersion spectroscopy (EDS) using an SU3500 SEM microscope (Hitachi, Tokyo, Japan) working alongside an UltraDry EDS Detector (ThermoFisher Scientific, Waltham, MA, USA) under the following conditions: acceleration voltage–15 keV, detector–BSE, scanning time–60 s, magnification ×50–×4000; the images were taken after spraying the sample with gold;
- Grain size and shape analysis by means of the optical method with image analysis using a G3S-ID analyser (Malvern Panalytical, Malvern, UK) under the following conditions: dispersion medium—air, sample amount—5 mm^3^, dispersion pressure—2.0 bar, dispersion time—20 ms, lens—×20, ×5, light—diascopic, automatically calibrated;
- The following parameters were analysed: width, length, CE diameter (the diameter of a circle with the same area as the projected area of the particle image), aspect ratio (the ratio of the width to the length of the particle), circularity (the ratio of the circumference of a circle equal to the object’s projected area to the perimeter of the object) [45].

#### 2.2.2. Physicochemical Methods of Separation

Size distribution was carried out via dry and wet sieving methods according to the standard [46] using a laboratory vibrating screen and standardised laboratory sieves (diameter—200 mm, height—50 mm, mesh size—0.025 mm, 0.045 mm, 0.063 mm, 0.1 mm and 0.2 mm) produced in accordance with the standard [47].

Float and sink analysis was performed according to the methodology provided in the standard [48]. A mixture of organic liquids (see Section 2.1) with densities of 1.4 g/cm^3^ to 2.5 g/cm^3^, increasing per 0.1 g/cm^3^, were used for the tests.

The flotation tests were carried out in a Mechanobr type flotation machine. Samples weighing about 100 g each were weighed, immersed in distilled water and left for about 24 h. Afterwards the samples were transferred quantitatively to a flotation chamber, where flotation was performed. The capacity of the chamber was 1 dm^3^, and the impeller rotations speed was 1200 rpm; after premixing the pulp, a reagent was added and the mixture was stirred for 1 min; the air supply was introduced afterwards and the foam product was collected until an empty foam appeared; the flotation products were filtered using filter paper (grade 292; Munktell, Finland), dried at a temperature of 105 °C in a laboratory dryer and weighed with an accuracy of ±0.01 g.

#### 2.2.3. Fertiliser Use Efficiency Assessment

Root growth assessment via cassette biotesting based on Phytotoxkit F™ (MicroBioTests, Gent, Belgium) was carried out. Root length measurement was performed with the ImageJ 1.46r software (Tiago Ferreira, Wayne Rasband; Public Domain, BSD-2) with an accuracy of ±0.01 mm.

Biomass growth assessment per the gravimetric method was carried out. The samples were dried at a temperature range of 40–50 °C in a laboratory dryer and weighed with an accuracy of ±0.01 g.

### 2.3. Soil Characterisation

The following was determined for the soil sample:

- Water extract pH by the potentiometric method per the standard [49]—leachability was performed according to the standard [50];
- Water extract metal content by means of inductively coupled plasma optical emission spectroscopy (ICP-OES);
- Carbon, hydrogen and total sulphur content by high-temperature incineration with IR detection;
- Organic carbon content by means of IR spectroscopy per the standard [51];
- Total nitrogen content by Kjehdahl’s method with Deward’s alloy addition;
- Phosphorus and potassium content by means of wavelength dispersive X-ray fluorescence spectroscopy (WDXRF);
- Polycyclic aromatic hydrocarbon (PAH) content by pressurised liquid extraction and high-performance liquid chromatography with fluorescence detection (HPLC-FLD) per the standard [52];
- Volatile aromatic hydrocarbon (WAH) content by headspace analysis and gas chromatography–mass spectrometry (GC–MS) per standard [53];

All the tests and analyses were performed at the Central Mining Institute’s Department of Environmental Monitoring.

### 2.4. Statistical Analysis

Statistical analysis of the experimental data was performed using Statistica 13.3 (StatsSoft, Kraków, Poland). Spearman’s rank correlation coefficient (*r*_s_) was calculated to indicates association between rank. The differences between many groups were compared using an analysis of variance one-way ANOVA and the post-hoc Tukey’s HSD (Honestly Significant Difference) test or the post-hoc Dunnett test. A probability level of *p* value less than 0.05 was considered statistically significant.

## 3. Results and Discussion

### 3.1. Characteristics of Ash from Poultry Manure Incineration

**Figure 1:**
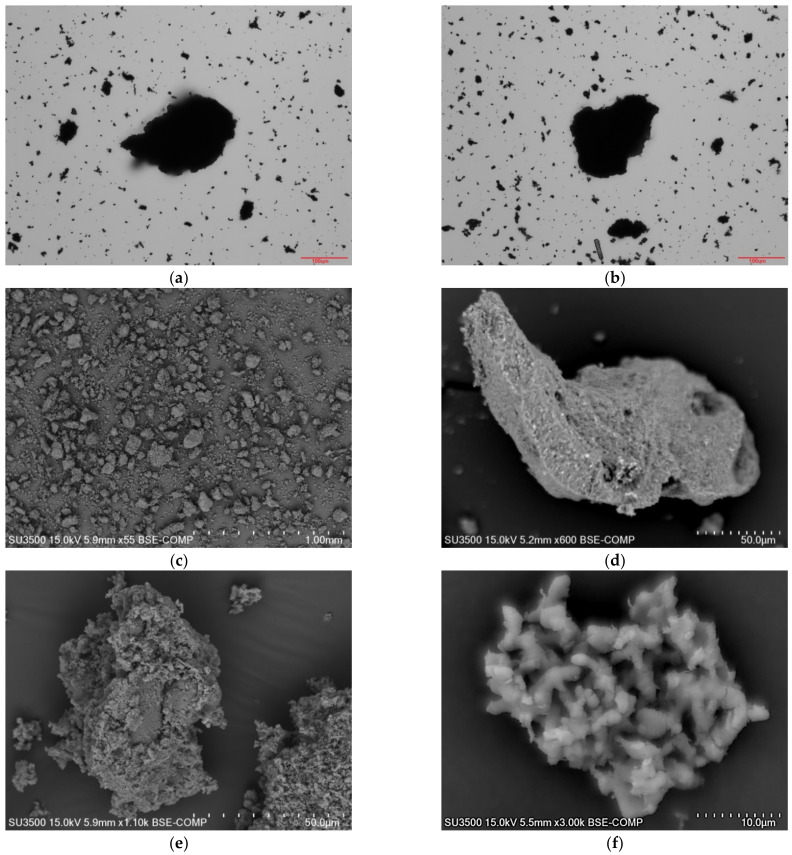
Images of selected grains for ash (sample PMA): (**a**,**b**) optical images; (**c**–**f**) SEM images.

The ash was varied in terms of particle size distribution, shape and surface morphology. The largest grains observed exhibited a length of 249 μm (Figure 1a,b) The majority of the grains exhibited irregular shapes, though spherical grains could be observed as well (Figure 1c–f). Grains characterized by a CE Diameter between 1–10 μm constituted 92.3%, 40.6% and 13.4% of the total, respectively by number, area and volume (Table 2). Ten percent of the tested grains were characterised by a CE Diameter under 1.28 μm. Fifty percent of the tested grains were characterised by a CE Diameter under 2.71 μm. Ninety percent of the tested grains were characterised by a CE Diameter under 8.86 μm. The average CE Diameter was 4.25 μm (Table 3).

**Figure 2:**
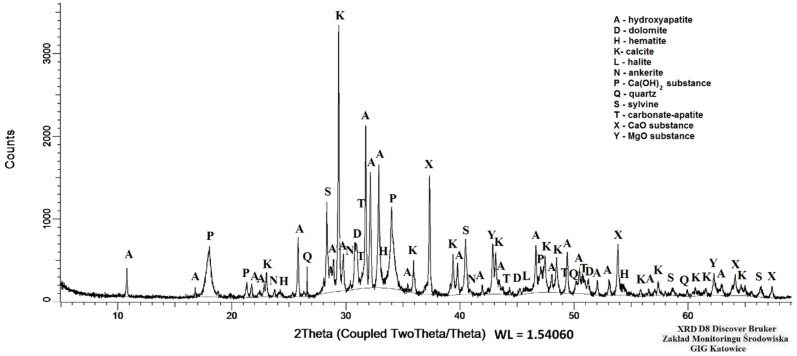
Ash diffraction photograph (sample PMA).

**Figure 3:**
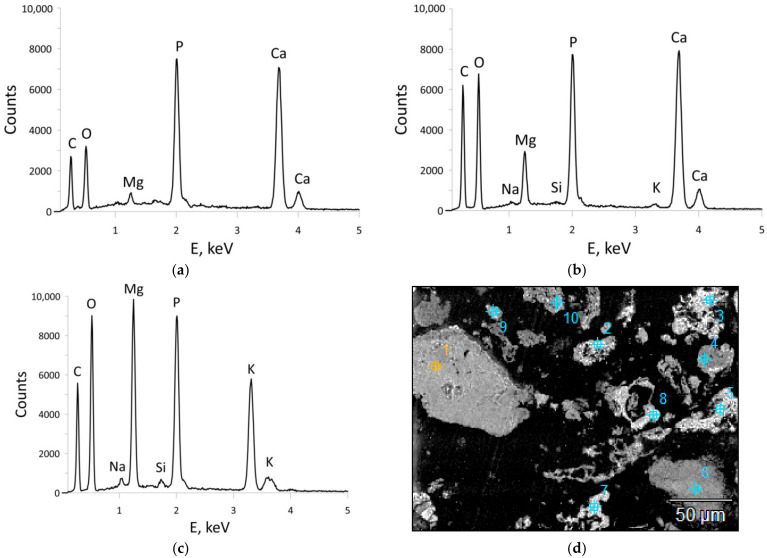
EDS spectra of the substance found in selected ash grains (sample PMA): (**a**) Ca-P substance; (**b**) Ca-Mg-P substance; (**c**) Mg-K-P substance; (**d**) view of analysed grains.

The main components of the ash were calcium (CaO 44.79 wt%) and phosphorus (P_2_O_5_ 15.73 wt%). The content of these elements was determined by two methods, i.e., XRF and ICP-OES. The difference between the results did not exceed 4.0% and 0.5%, respectively for the determination of calcium and phosphorus. The following components were present in quantities of several percent: potassium (K_2_O 6.75 wt%), magnesium (MgO 6.36 wt%), sulphur (SO_3_ 4.72 wt%) and chlorine (Cl 3.92 wt%). In terms of trace elements, attention should be drawn to the high content of manganese (Mn 1040 ppm), zinc (Zn 1310 ppm) and strontium (Sr 582 ppm). The loss on ignition at the temperatures of 815 °C was 14.86 wt% (Table 4). The total carbon, total sulphur and total hydrogen content equalled 2.47 wt%, 1.78 wt%, 0.14 wt%, respectively. The nitrogen content in the tested sample was below the determination limit (<0.15 wt%). The presence of carbonates (calcite, dolomite, carbonate apatite and ankerite) and chlorides (sylvine, halite) in the tested ash was confirmed by mineralogical testing. Phosphorus in crystalline form occurred together with calcium, primarily as hydroxyapatite (18.0 wt%) and carbonate apatite (4.0 wt%). However, no presence of whitlockite was identified. Besides the crystalline minerals, the tested ash also contained 18.5 wt% of an amorphous substance. The ratio of the crystalline phase to the amorphous phase calculated on the basis of weight proportions was 4.4 (Table 5, Figure 2). Phosphorus was also a component of the amorphous substance and occurred together with calcium as well as potassium and magnesium (Figure 3).

### 3.2. Characteristics of Anthropogenic Land Soil

Anthropogenic land soil (SW) contained phosphorus in the amount below 0.1 wt%, nitrogen–below 0.2 wt% and potassium in the amount of 2.0 wt%. The soil contained 1.73 mg/kg of polycyclic aromatic hydrocarbons (Table 6). The soil also contained metals, mainly manganese and barium (Table 7).

### 3.3. Physicochemical Methods of Phosphate Concentrate Separation from Ash

The following physicochemical methods were applied to separate the phosphate concentrate from ash generated from poultry manure incineration: (i) methods based on particle size distribution (size analysis by wet and dry sieving); (ii) a method based on grain wettability difference (flotation); and (iii) a method based on grain density difference (float and sink analysis).

Independent sieving cycles of ash (PMA) were carried out by the wet and dry methods (Table 8 and Table 9). The P_2_O_5_ content in individual fractions obtained by dry sieving ranged within 15.27–16.35 wt%, and CaO–within 44.46–50.05 wt%. Therefore, dry sieving did not make it possible to separate a fraction with a significantly greater phosphorus content.

The ash grain composition, obtained by wet sieving, was typical for polydispersive materials, i.e., grain weight yield increased together with the increase in fragmentation. The P_2_O_5_ content in +0.025 mm fractions (PC) was similar and ranged within 23.56–27.29 wt%. The P_2_O_5_ content in the −0.025 mm fraction was much lower and amounted to 15.07 wt%. The CaO content ranged within 38.71–50.64 wt% and was the highest in the −0.025 mm fraction. Size grading by wet sieving at a particle size of 0.025 mm can be considered as a method for phosphate concentration. The average P_2_O_5_ content in +0.025 mm fractions was 24.56 wt%, and its total weight yield was 34.7%. The phosphorus content in the +0.025 mm fraction was 1.6 times greater compared to the −0.025 mm fraction. Therefore, grains varied in size and there was a positive correlation between phosphorus content and grain size (*r*_s_ = 0.94, *p* < 0.05).

**Figure 4:**
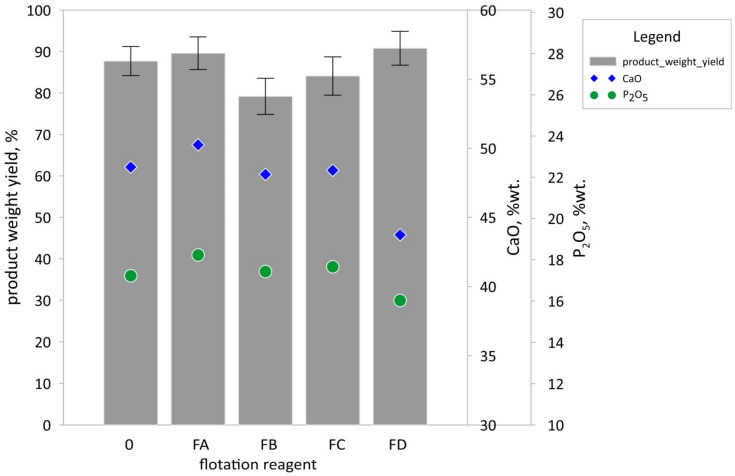
Flotation test results, and phosphorus and calcium contents in separated fractions. Explanations: 0–test without flotation reagent; flotation reagent symbols according to Table 1; bar–product weight yield mean value; whiskers–standard deviation; blue diamond–CaO content mean value; green circle–P_2_O_5_ content mean value (n = 3).

Four reagents with various chemical compositions and properties were used for the flotational phosphorus compound concentration from ash suspensions (Table 1). The suspension exhibited a certain natural flotation action, without the addition of reagents. The product weight yields in all the experiments were relatively high and ranged within 79.2%–90.8%. Adding reagents resulted in only slight increases in weight yield in experiments with reagents FR-A and FR-D, while decreasing it in experiments with reagents FR-B and FR-C. P_2_O_5_ content in the concentrates ranged from 16.03 wt% to 18.23 wt%, and CaO content was from 43.74 wt% to 50.25 wt%. The applied reagents were nonselective and resulted in no significant increase in phosphorus content in the products (Figure 4).

Float and sink tests revealed that the ash grains, regardless of size and shape, were practically uniform in terms of density. The density of all the grains ranged from 2.4 g/cm^3^ to 2.5 g/cm^3^, which made it practically impossible to separate the grains by density.

### 3.4. Use of Ash from Poultry Manure Incineration as a Substitute for Phosphatic Fertiliser

The goal of the tests was to investigate the usefulness of the obtained phosphate concentrate (PC) with a P_2_O_5_ content of 24.56 wt%, separated from ash generated from poultry manure incineration, as a fertiliser for grass cultivation in rough and degraded land. Technological effectiveness of the process through its verification in pot and field tests was determined.

#### 3.4.1. Cassette Biotesting

The first stage of the tests employed a cassette biotesting method. Generally, biotesting is applied to inspect a plant’s reaction (germination, seed and root growth) to harmful or toxic substances in the soil. As phosphorus is an element necessary for correct plant development, particularly during germination and root system growth, using biotests in the first stage of growth is suitable for inspecting the plant’s reaction to the diverse doses of the tested fertiliser product, particularly in comparison with commercial fertilisers. Selecting the appropriate dose of the fertiliser is important, as introducing quantities of phosphorus greater than the plant’s nutritional needs can be justified only under the conditions of cultivation in soils poor in phosphorus. Otherwise, this results in its undesired accumulation in the soil and excessive leaching to waters.

**Figure 5:**
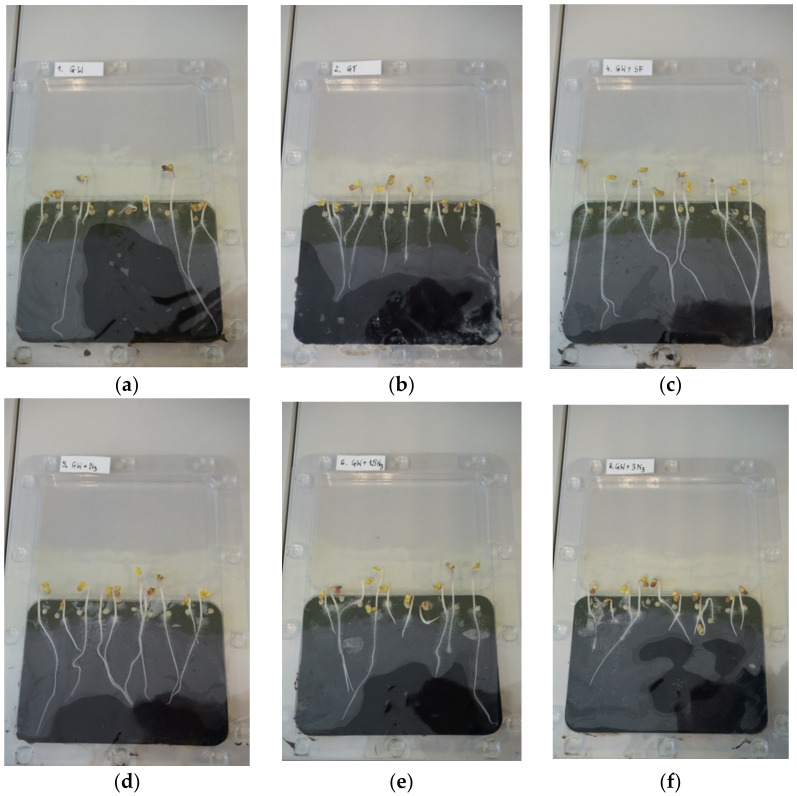
Cassette cultivation–view after 72 h: (**a**) root growth in test C1; (**b**) root growth in test L; (**c**) root growth in the C2; (**d**) root growth in test C4; (**e**) root growth in test C5; (**f**) root growth in test C7–visible variable germination and root growth of white mustard *Sinapis alba* L. Explanations: test symbols according to Table 11.

Twenty-two substrates were prepared, composed of soil collected from anthropogenic land (SW) or reference soil (SR) and fertiliser, i.e., the phosphate concentrate (PC), the unprocessed ash (sample PMA) or commercial fertiliser (CF-S, CF-A). For comparison purposes, the test was also conducted in a substrate composed only of soil. The initial dose of the fertiliser was defined based on doses adopted for commercial fertilisers and on the bioavailable phosphorus content determined using citric acid (Table 10). The plant material used for the tests constituted selected seeds of white mustard *Sinapis alba* L. Ten seeds were placed in each cassette. The test was repeated three times for each substrate. Tests were conducted for 72 h; afterwards the root growth was measured (Table 11, Figure 5).

**Figure 6:**
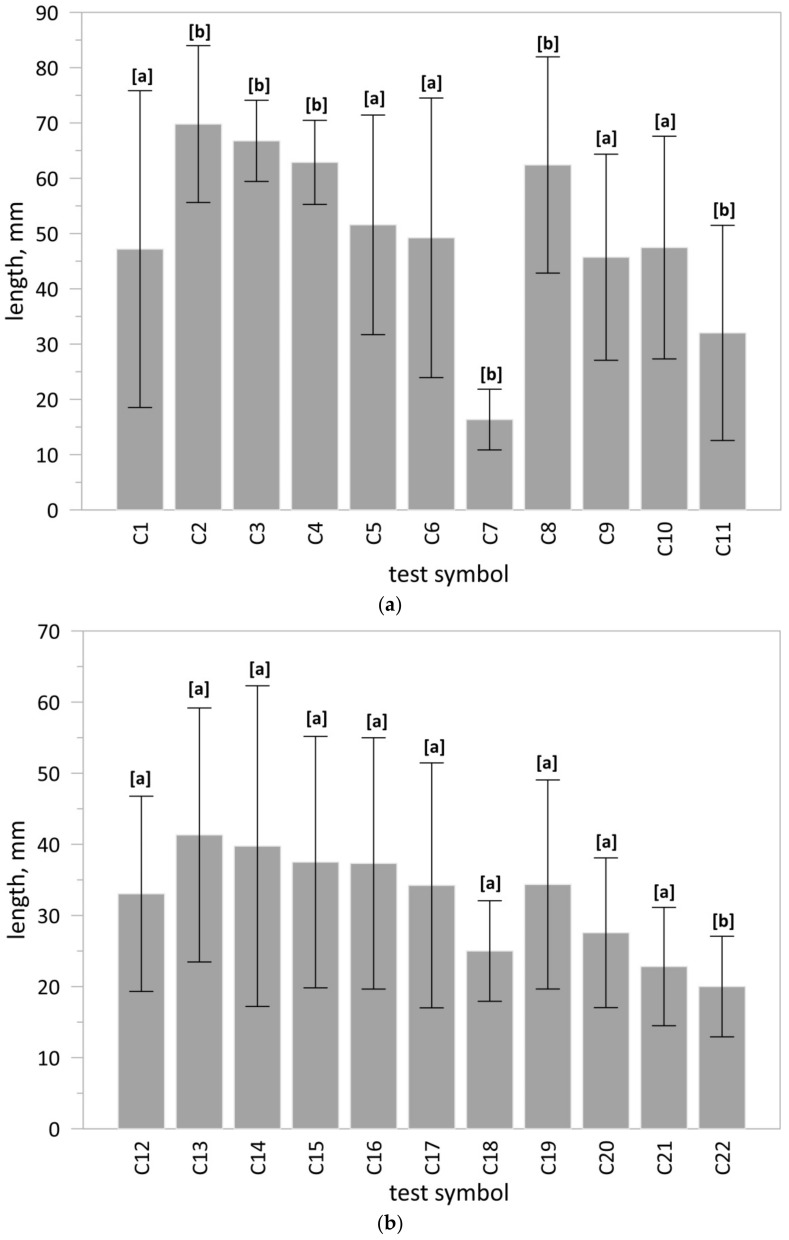
Root growth in cassette biotests: (**a**) anthropogenic land soil (SW); (**b**) reference soil (SR). Explanations: bar–mean value, whiskers–standard deviation (n = 24); test symbol according to Table 11; significant differences (if letter b) or no significant differences (if letter a) between reference group (C1 or C12) and other substrates according to the Dunnett test.

The length of roots obtained in tests with the use of raw ash (PMA) or the obtained phosphorus concentrate (PC) in the basic dose was comparable to the results obtained with the use of commercial fertilisers (CF-S, CF-A). Increasing the dose reduced their growth (Figure 6). The reference soil (SR) was almost completely free of organic matter. In anthropogenic soil (SW), there were, apart from impurities, elements that can determine the growth of the roots and the above-ground part of plant. A faster root growth rate on anthropogenic soil then on reference soil was observed.

The results of the one-way ANOVA test indicated that at least one group of substrates differed from the other (*p* < 0.05). In the next step, the Dunnett test was conducted to clarify which groups differ from reference test (Figure 6).

#### 3.4.2. Pot Cultivation

**Figure 7:**
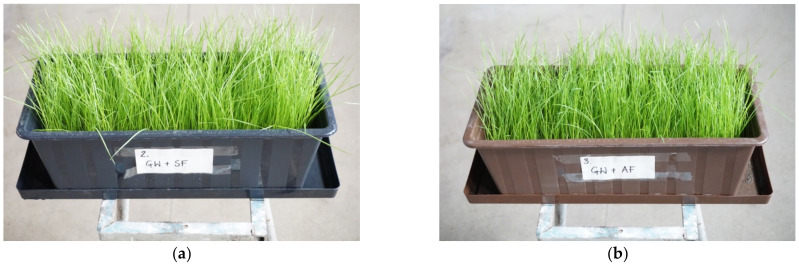

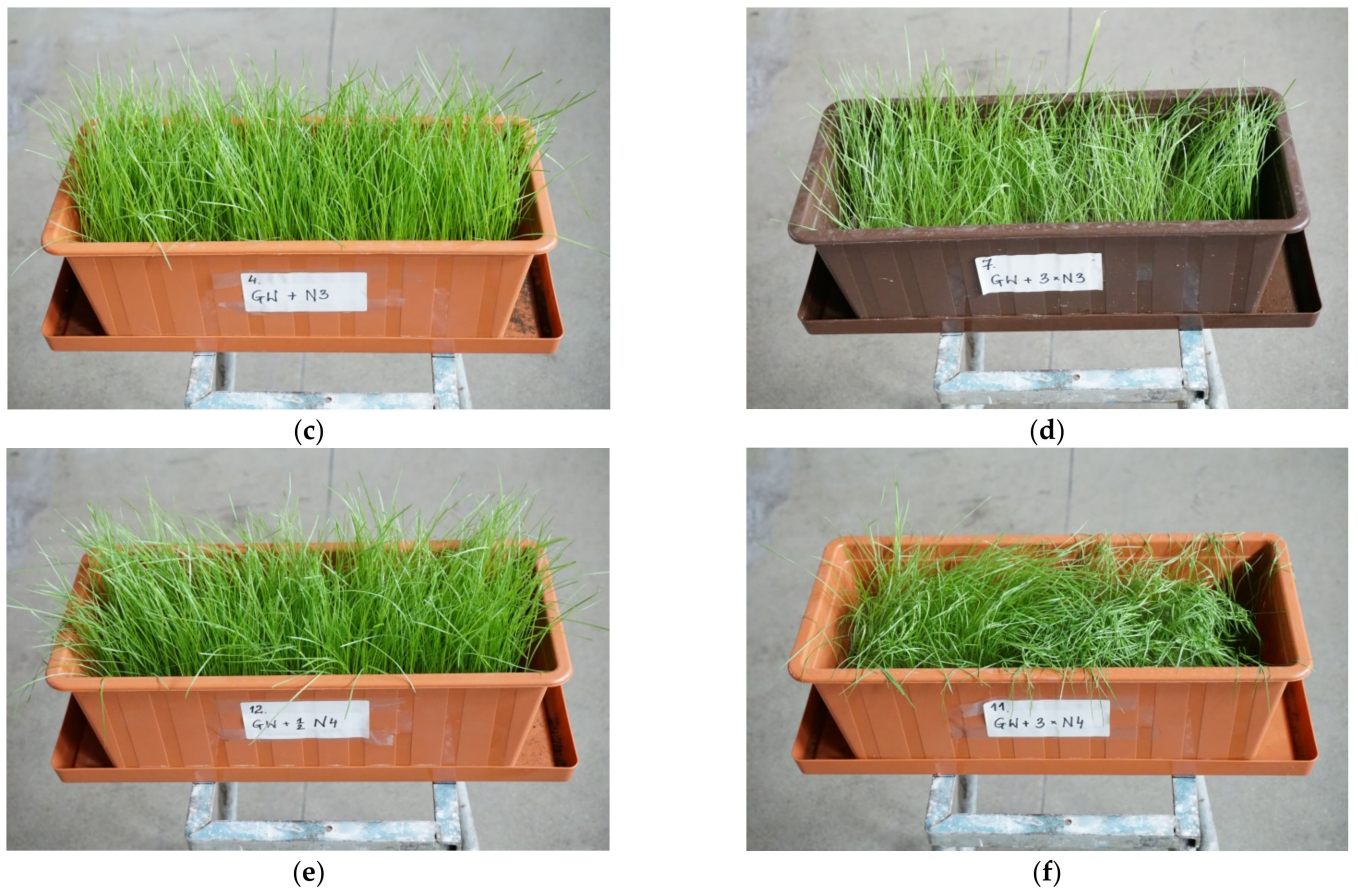
Pot cultivation–view on the 17th day from the start of cultivation: (**a**) biomass growth in test P2; (**b**) biomass growth in test P3; (**c**) biomass growth in test P5; (**d**) biomass growth in test P8; (**e**) biomass growth in test P9; (**f**) biomass growth in test P13–visible different height and condition of sown grasses. Explanations: test symbol according to Table 12.

The following was recommended for the next stage, i.e., pot cultivation: substrates with the phosphate concentrate (PC) and the raw ash (PMA) in the basic doses, increased by half, double and triple, as well as with commercial fertilisers for comparison. Two new substrates were also proposed, with half doses of the tested product or raw ash. Therefore, 13 substrates were prepared, composed of anthropogenic land soil (SW) and fertilisers in the appropriate doses (Table 12). The substrate was sown with a universal mixture of grass for rough and dry land (Table 1) at a dose of 3 g per pot. The cultivation was conducted for 36 days at a stable temperature (16–18 °C). The cultivation was exposed to sunlight and systematically watered. Varied germination and water absorption was observed, as well as varied grass condition, colour, turgor and height (Figure 7). After cultivation was concluded, the epigeal parts of the plants were collected from all the pots, dried and weighed, and biomass growth was determined.

**Figure 8:**
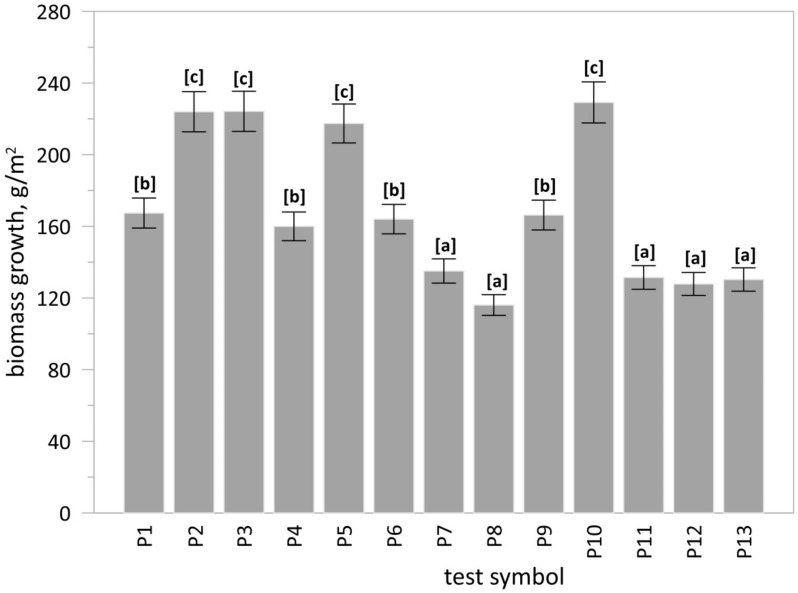
Plant biomass growth in pot cultivation. Explanation: bar–mean value, whiskers–standard deviation (n = 3); test symbol according to Table 12; different letters [a, b, c] indicate significant differences among groups (substrates) according to the Tukey’s HSD test.

On the substrate without the use of fertiliser, the biomass growth was 167 g/m^2^. The greatest biomass growth, i.e., 229 g/m^2^ was observed in the substrate fertilised using raw ash (PMA) in the basic dose. Both the reduction of the base dose by half and its increase significantly decreased the biomass growth. Very similar results, i.e., 224 g/m^2^, were noted for substrates subjected to commercial fertilisers. The biomass growth observed for the substrate subjected to the phosphate concentrate (PC) in the basic dose was 217 g/m^2^. Applying half the basic dose of the phosphate concentrate or increasing it yielded lower growth (Figure 8).

The results of the one-way ANOVA test indicated that at least one group of substrates differed from the other (*p* < 0.05). In the next step, the Tukey’s HSD test was conducted to clarify which groups differ from each other (Figure 8).

#### 3.4.3. Field Cultivation

**Figure 9:**
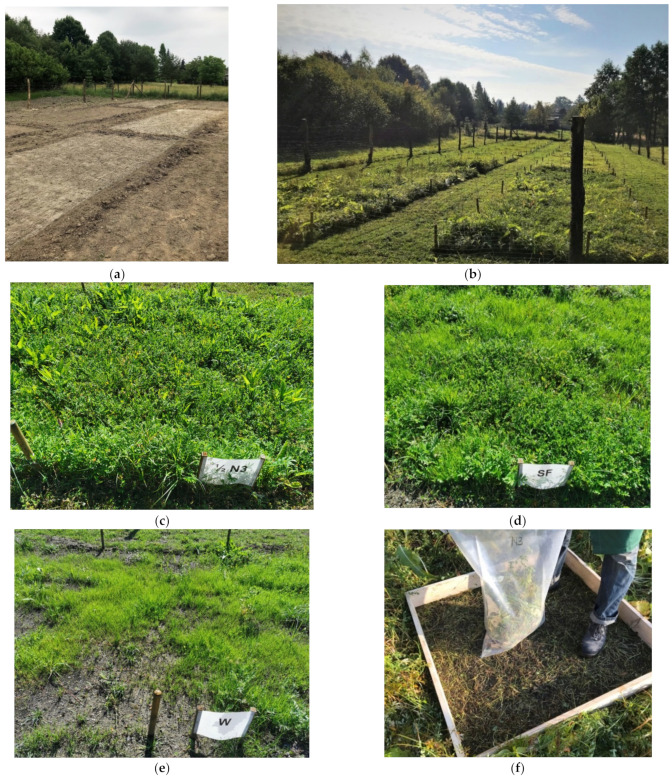
Field cultivation: (**a**) sown plants; (**b**) biomass growth in the plots; (**c**) biomass growth in test F4; (**d**) biomass growth in test F3, (**e**) biomass growth in test F1, (**f**) biomass collection from each plot. Explanation: test symbol according to Table 13.

Field tests were conducted at a testing ground established on anthropogenic land, at the base of the “Waleska” coal waste dump in Łaziska Górne (Poland). The testing ground was established on flat land, in the area of the discharge water reservoir from the waste dump. Originally, this area was covered in ruderal vegetation originating from spontaneous succession. It was dominated by high, dense and strongly expansive grass–bushgrass *Calamagrostis epigejos* (L.) as well as a small community of Canadian goldenrod *Solidago canadensis* L. The testing ground with an area of 700 m^2^ was fenced off, and agricultural preparatory action was taken, consisting in topsoil ploughing and local vegetation biomass removal. Ten cultivation plots with an area of 24 m^2^ each were established at the testing ground. Using a semi-automatic seeder, a layer of fertilisers in doses determined based on the pot cultivation was spread on nine plots. To monitor the differences in plant growth and condition, the fertiliser layer was not applied to one of the plots–the reference plot (Table 13). After 14 days, the mix of grass was sown to an amount of 0.5 kg for each plot (Table 1). The testing ground cultivation was conducted for 4 months. The plots were not watered artificially (Figure 9b–e). After the cultivation the epigeal part of the biomass was collected, which was afterwards dried and weighed (Figure 9f).

The biomass growth in the reference plot was 344 g/m^2^. The greatest biomass growth i.e., 433 g/m^2^ was observed in plots with the commercial fertiliser Azofoska. This fertiliser provides complex substrate enrichment in nitrogen, potassium and phosphorus.

**Figure 10:**
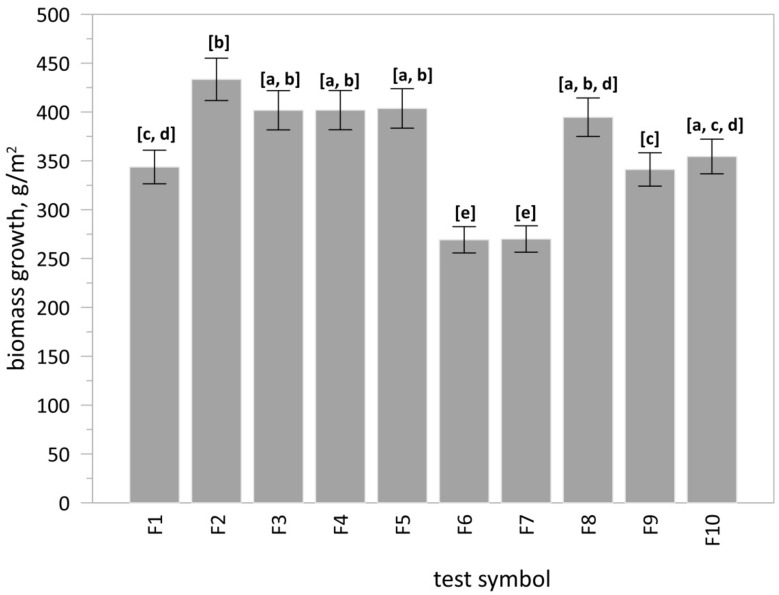
Plant biomass growth in field cultivation. Explanation: bar–mean value, whiskers –standard deviation (n = 3); test symbol according to Table 13; different letters [a, b, c, d, e] indicate significant differences among groups (plots) according to the Tukey’s HSD test.

Very good biomass growth results were obtained in plots subjected to the phosphorus concentrate (PC) in both the basic dose (404 g/m^2)^ and half the basic dose (402 g/m^2^). The yield from these plots was comparable to the yield with the commercial fertiliser Superfosfat (402 g/m^2^). Applying double the basic dose considerably lowered the biomass growth to 269 g/m^2^. Using unprocessed ash (PMA) at half the basic dose also provided a slightly lower yield than the commercial fertiliser Superfosfat (Figure 10).

The results of the one-way ANOVA test indicated that at least one group of plots differed from the other (*p* < 0.05). In the next step, the Tukey’s HSD test was conducted to clarify which groups differ from each other (Figure 10).

Apart from the sown mix of grass, the natural seed and underlying rhizome bank was activated in all the plots. The compositions of species observed were very similar in all the plots. Apart from the sown grass, the following species exhibited significant contributions: silverweed *Potentilla anserina* L., common sorrel *Rumex acetosa* L., common comfrey *Symphytum officinale* L., broadleaf plantain *Plantago major* L., petty spurge *Euphorbia peplus* L., and white clover *Trifolium repens* L. These species were not present during pot cultivation, as the anthropogenic soil used in the said cultivation was first sifted, which is a process that eliminates species which reproduce by stolons to a major degree. The better availability of light during field cultivation is a factor that also results in seed bank activation. In the original state, the aforementioned species were not found on the land in question, as the entire area was covered with high, dense and strongly expansive grass–*bushgrass Calamagrostis epigejos* L.

The soil for pot cultivation was derived from an anthropogenic area (the area of later field cultivation) and prepared according to the method described in the Materials section. Thus, it was deprived of plant rhizomes, tubers and diasporas, which constituted a natural “seed bank”. In the P5 pot culture, the base dose of phosphorus concentrate was used, i.e., the one with the content of bioavailable phosphorus comparable to that contained in the dose recommended by the manufacturer of commercial fertilisers. Half of the base dose (P4) proved insufficient and resulted in an increase of the sown grasses biomass comparable to test P1. Increasing the concentrate dose (P6, P7, P8) resulted in smaller increments and, therefore, the phosphorus dose was optimally selected for test P5, which translated into biomass increments comparable to commercial fertilisers. A similar relationship was observed for raw ash (P9, P10, P11, P12, P13).

The soil in the plots of field cultivation contained a “seed bank”. At the same time, despite the use of protective belts, other plant species were deposited by wind and water. The pot culture conditions were constant (humidity, temperature, sunlight) and isolated from natural factors. In the plots where the concentrate was applied, half of the base dose (F4) was sufficient to cause the biomass increase comparable to the plot where Superfosfat (F3) was used. This increase indicates that half of the dose of the phosphorus coconcentrate can be used for fertilization. The raw ash used as a fertiliser in the plots of plots F8, F9, F10 showed a slight odour under the cultivation conditions, similarly to the pot cultivation.

A comparison of biomass increments in pot and field cultivations showed that they were much higher in field cultivation. All grass species were characterized by better condition, turgor and colour. The invasion and the “seed bank” also gave rise to other plants, which enriched the species-related phytocoenosis.

Attempts at an agricultural use of poultry manure or litter ash were undertaken by numerous researchers [54,55,56,57,58,59,60,61,62,63,64,65,66]. Their results present this material as an interesting fertiliser, as it contains both of the necessary nutrients: phosphorus and potassium, found in compounds exhibiting good bioavailability in pot and field tests. The ash exhibits efficiency similar to reference mineral fertilisers such as: triple superphosphate, dicalcium phosphate, potassium chloride and potassium sulphate [57,58,59].

In their research, Vance et al. [60,61] showed a similar poultry litter ash (PLA) fertilization efficiency in relation to triple superphosphate, i.e., the use of PLA enabled obtaining comparable yields (biomass increase). The research revealed that phosphorus from PLA is less soluble in water in comparison with that from triple superphosphate, which means that the use of PLA allowed the avoidance of phosphorus loss as a result of soil leaching.

Codling et.al [62,63] compared the fertilization efficiency of PLA in relation to potassium phosphate and superphosphate in the cultivation of the following plants: wheat (*Triticum aestivum* L.), peanut (*Arachis hypogaea* L.) and soybean (*Glycine max* L.) PLA was also characterized by similar effectiveness to these fertilisers. In acidic soils, the use of PLA resulted in higher wheat yields. This effect did not occur when limestone was used to regulate soil pH. In the aforementioned case, for soil with an initial pH of 5.42, the application of PLA increased pH and an increase in the obtained biomass. In the case under consideration, the initial pH was 6.7.

Pagliari et al. [64] in their research on the use of ash obtained from turkey manure in the cultivation of corn (*Zea mays* L.) reported a decrease in biomass yield compared to the alternative superphosphate and KCl mixture. The research was carried out on soil with an initial pH of 7.0, and it was found that a further increase in the dose causes a gradual decrease in the effectiveness of both fertilisers, which was also confirmed by these studies.

The PLA application could decrease the biomass yield due to the risk of increasing the pH, reducing the availability of nutrients [65]. According to the literature, the application of superphosphate decreases pH. In the case of soils with a relatively high pH, the use of PLA may reduce the biomass yield in relation to soils where commercial fertilisers such as superphosphate have been applied.

In their research on the use of PLA in the cultivation of phacelia (*Phacelia tanacetifolia* Lisette), buckwheat (*Fagopyrum escultentum* Lifago), oil radish (*Raphnus sativus oleiformis* Adagio), and ryegras (*Lolium multiflorum* westerwoldicum Gordo), Bachmann et al. [66] obtained a biomass increase at a level similar to or higher than that in the case of potassium phosphate. The content of phosphorus in the soil fertilized with PLA was similar to that fertilized with potassium phosphate in all tested fractions.

## 4. Conclusions

P-rich waste, including ashes produced in the process of poultry manure incineration, can be treated as raw materials to be used in other sectors of the economy in accordance with the circular economy promoted by the EU. The effectiveness of its use as a substitute for a phosphorus fertiliser has been confirmed by laboratory and field tests. The conducted research allowed the development of a methodology for managing ash from poultry manure incineration by separating the useful fraction (enriched with phosphorus) and, next, making a cultivation substrate based on it. The results of these studies provided a basis for the invention [67].

The P_2_O_5_ content in the ash from poultry manure incineration and the obtained product by wet sieving was, respectively, 15.73 wt% and 24.56 wt%. The fertiliser dose had a significant influence on the biomass growth. The basic dose of tested materials was defined based on doses adopted for commercial fertilisers and on the bioavailable phosphorus content determined using citric acid.

The studies aimed at separating a phosphate concentrate from ash allowed the following conclusions to be made:

1. Grains of the ash from poultry manure incineration are practically uniform in terms of density, which made it almost impossible to separate the grains by the float and sink method.
2. The grains were characterised by different wettability, but the phosphorus content in the fractions separated by flotation was similar.
3. The grains varied in size and there was a positive correlation between phosphorus content and grain size.
4. Size grading by wet sieving at a particle size of 0.025 mm can be considered a method for phosphate concentration.

The studies aimed at assessing the fertilisation efficiency of ash material allowed the following conclusions to be made:

1. Both the raw ash from poultry manure incineration and the separated phosphorus concentrate can be used as a substitute for commercial fertiliser.
2. Cassette biotests allowed the doses of fertilisers used to be optimised.
3. In pot cultivation, the greatest biomass growth, i.e., 229 g/m^2^, was found on the substrate, where raw ash was used as a fertiliser at a dose of 330 g/m^3^. The biomass growth for the substrate, where the separated phosphorus concentrate was used in the dose of 190 g/m^3^, was 217 g/m^2^. Both reducing or increasing these doses significantly decreased the biomass growth. The obtained results were compared with those for commercial fertilisers, where the biomass growth was 224 g/m^2^.
4. In field cultivation, the application of the obtained phosphorus concentrate at a dose of 95 g/m^3^ had a comparable yield to the use of commercial fertiliser at a dose of 75 g/m^3^. Unprocessed ash had to be used in larger amounts, i.e., 165 g/m^3^ to have a comparable yield to commercial fertiliser.

## Figures and Tables

**Table 1:** Materials used in the tests.

Symbol	Description
FR-A	Cationic surfactants: quaternary ammonium compounds and ethoxylated and quaternised fatty amine and amphoteric amines from the group of betaines, a mixture of dioxane alcohols.
FR-B	Betaine-type amphoteric surfactants, wetting additives, a mixture of dioxane alcohols.
FR-C	Non-ionic surfactants, auxiliaries in the form of block copolymers of ethylene oxide and propylene oxide and wetting additives, 2-ethyl hexyl alcohol and a mixture of dioxane alcohols.
FR-D	Hydrocarbons from petroleum processing with added surfactants.
SR	OECD artificial soil formed mainly from mineral material, according to the standard [40].
CF-S	“Superfosfat” enriched mineral fertiliser (Pro-Agro). Granular fertiliser (2 mm–5 mm). Composition: 40 wt% P_2_O_5_, 34 wt% CaO, 5 wt% SO_3_, with added micro-elements [41].
CF-A	“Azofoska” universal garden fertiliser (Grupa INCO). Composition: 13.3 wt% N, 6.1 wt% P, 17.1 wt% K, 4.5% MgO, 21.0 wt% SO_3_, 0.025 wt% B, 0.09 wt% Cu, 0.14 wt% Fe, 0.14 wt% Mn, 0.02 wt% Mo, 0.025 wt% Zn [42].
MG	Mixture of grass in the following mass ratios: 65% *Lolium perenne* L., 5% *Poa pratensis* L., 25% *Festuca rubra* L., 5% *Festuca trachyphylla* K.

**Table 2:** Number, area and volume particle size classification (according to the CE Diameter parameter) of the ash (sample PMA).

Classification	Grain Class, μm							
	1–10	10–20	20–30	30–40	40–50	50–100	100–150	>150
Number, %	92.35	6.64	0.71	0.18	0.07	0.04	0.01	0.00
Area, %	40.62	31.97	11.03	5.83	3.65	5.90	0.65	0.35
Volume, %	13.43	24.09	14.44	10.85	8.78	21.29	3.78	3.34

**Table 3:** Number particle size and shape distribution of the ash (sample PMA).

Parameter ^(^^a)^	Value	Parameter	Value
CE Diameter D [n, 0.1], μm	1.28	Aspect Ratio D [n, 0.1]	0.553
CE Diameter D [n, 0.5], μm	2.71	Aspect Ratio D [n, 0.5]	0.777
CE Diameter D [n, 0.9], μm	8.86	Aspect Ratio D [n, 0.9]	0.906
CE Diameter Mean, μm	4.25	Aspect Ratio Mean	0.764
Width D [n, 0.1], μm	1.34	Circularity D [n, 0.1]	0.764
Width D [n, 0.5], μm	2.53	Circularity D [n, 0.5]	0.936
Width D [n, 0.9], μm	8.11	Circularity D [n, 0.9]	0.983
Width Mean, μm	3.97	Circularity Mean	0.909
Length D [n, 0.1], μm	1.53		
Length D [n, 0.5], μm	3.36		
Length D [n, 0.9], μm	12.52		
Length Mean, μm	5.73		

^(a)^ Explanations: parameter definition–see Section 2.2.1.

**Table 4:** Chemical composition of ash from poultry manure incineration (sample PMA).

Main Elements	Content ^(^^c)^, wt%	Trace Elements	Content ^(^^c)^, ppm
SiO_2_	1.61 ± 0.10	Ag	<2
Al_2_O_3_	0.26 ± 0.02	As	<2
Fe_2_O_3_	0.43 ± 0.02	Ba	72 ± 14
CaO	44.79 ^(a)^ ± 4.48 46.59 ^(b)^ ± 9.32	Cd	7 ± 3
MgO	6.36 ± 0.64	Co	6 ± 3
Na_2_O	3.48 ± 0.42	Cr	<2
K_2_O	6.75 ± 0.68	Cu	<2
TiO_2_	0.020 ± 0.002	Mo	<2
P_2_O_5_	15.73 ^(a)^ ± 2.20 15.80 ^(b)^ ± 3.16	Ni	8 ± 3
SO_3_	4.72 ± 0.71	Pb	49 ± 10
MnO	0.13 ± 0.03	Rb	<2
ZnO	0.16 ± 0.03	Sb	<2
LOI	14.86 ± 2.23	Sn	<2
Other	0.69	Sr	582 ± 116
Sum	100.00	V	<2

Explanations: LOI–loss on ignition at 815 °C, ^(a)^ XRF method, ^(b)^ ICP-OES method, ^(c)^ content ± expended uncertainty, coverage factor of 2 and significance level of 95%.

**Table 5:** Phase composition of ash (sample PMA).

Chemical Formula	Content, wt%	Chemical Formula	Content, wt%
CaCO_3_	24.5	Ca_5_(PO_4_,CO_3_,OH)_3_	4.0
Ca_5_(OH)(PO_4_)_3_	18.0	KCl	4.0
CaO	7.0	SiO_2_	2.5
Ca(OH)_2_	6.5	CaFe(CO_3_)_2_	2.0
CaMg(CO_3_)_2_	5.5	Fe_2_O_3_	2.0
MgO	5.0	NaCl	0.5
Total phosporous crystalline phase, %wt.		22.0	
Total crystalline phase, %wt.		81.5	
Total amorphous phase, %wt.		18.5	

**Table 6:** Physicochemical characteristics of anthropogenic land soil (sample SW).

Parameter	Content ^(c)^	Unit
Σ PAH ^(a)^	1.73 ± 0.44	mg/kg
Σ WAH ^(b)^	<0.080	mg/kg
Ash	90.56 ± 3.62	wt%
TIC	<0.10	wt%
TOC	3.78 ± 0.30	wt%
S	0.04 ± 0.01	wt%
N	0.17 ± 0.02	wt%
P	0.067 ± 0.009	wt%
K	2.01 ± 0.20	wt%

Explanations: TIC–total inorganic carbon; TOC–total organic carbon; ^(a)^ polycyclic aromatic hydrocarbon: naphthalene, acenaphthalene, fluorene, phenantrene, anthracene, fluoranthen, pyrene, benzo(a)anthracene, chrysene, benzo(b)fluoranthen, benzo(k)fluoranthen, benzo(a)pyren, dibenzo(a,h)anthracen, benzo(g,h,i)perylene, indeno(1,2,3-cd)pyren; ^(b)^ volatile aromatic hydrocarbon: benzene, toulene, ethylbenzene, m- and p-xylene, o-xylene and styrene; ^(c)^ content ± expended uncertainty, coverage factor of 2 and significance level of 95%.

**Table 7:** Content of metals in the anthropogenic land soil water extract ^(a)^ (sample SW).

Parameter	Content ^(b^^)^, ppm	Parameter	Content ^(b^^)^, ppm
As	16 ± 3	Mo	1.0 ± 0.3
Ba	309 ± 62	Ni	21 ± 4
Cd	1.0 ± 0.3	Pb	49 ± 10
Co	13 ± 3	Sb	1.0 ± 0.3
Cr	37 ± 7	Se	2.0 ± 0.7
Cu	23 ± 5	Sn	3 ± 1
Hg	0.20 ± 0.06	Zn	147 ± 29
Mn	1510 ± 302		

Explanations: ^(a)^ water extract pH 6.7; ^(b)^ content ± expended uncertainty, coverage factor of 2 and significance level of 95%.

**Table 8:** Dry grain analysis results, and phosphorus and calcium contents in separated fractions.

Class Grain, mm	Weight Yield, %	Total Yield, %	P_2_O_5_ ^(a^^)^ Content ^(b^^)^, wt%	CaO ^(a^^)^ Content ^(b^^)^, wt%
+0.2	4.7	4.7	15.48	50.05
0.2–0.1	12.0	16.7	15.48	48.86
0.1–0.063	19.9	36.6	16.35	47.47
0.063–0.045	27.8	64.4	15.76	45.96
0.045–0.025	28.9	93.3	15.43	45.67
−0.025	6.7	100.0	15.27	44.56
Sum	100.0		15.71	46.62

Explanations: ^(a)^ ICP-OES method, ^(b)^ expended uncertainty 20%, coverage factor of 2 and significance level of 95%.

**Table 9:** Wet grain analysis results, and phosphorus and calcium contents in separated fractions.

Class Grain, mm	Weight Yield, %	Total Yield, %	P_2_O_5_ ^(a^^)^ Content ^(b^^)^, wt%	CaO ^(a^^)^ Content ^(b^^)^, wt%
+0.2	1.8	1.8	27.30	45.02
0.2–0.1	5.3	7.1	26.77	38.71
0.1–0.063	8.6	15.7	24.59	39.62
0.063–0.045	1.7	17.4	24.80	41.12
0.045–0.025	17.3	34.7	23.56	44.86
−0.025	65.3	100.0	15.07	50.64
Sum	100.0		18.36	47.80

Explanations: ^(a)^ ICP-OES method, ^(b)^ expended uncertainty 20%, coverage factor of 2 and significance level of 95%.

**Table 10:** Content of total phosphorus (P_2_O_5_) and bioavailable phosphorus (P_2_O_5_,_CA_) in the tested samples, and the defined basic doses (DB) of the fertiliser.

Parameter	Sample			
	PMA	PC	CF-S	CF-A
P_2_O_5_, wt%	15.80	24.56	40.00 [38]	6.80 [39]
P_2_O_5,CA_, wt%	6.89	11.85	30.65	6.52
DB, g/m^3^ substrate ^(^^a)^	330	190	75	150

Explanations: PMA–ash from poultry manure incineration, PC–separated phosphate concentrate, CF-S–commercial fertiliser Superfosfat, CF-A–commercial fertiliser Azofoska, ^(^^a)^ in the case of field cultivation the calculation was based on a layer with a thickness of 0.2 m.

**Table 11:** Substrate preparation for cassette biotesting.

Test Symbol	Substrate			Test Symbol	Substrate		
	Soil	Fertiliser			Soil	Fertiliser	
		Type	Dose			Type	Dose
C1	SW	-	-	C12	SR	-	-
C2	SW	CF-S	DB	C13	SR	CF-S	DB
C3	SW	CF-A	DB	C14	SR	CF-A	DB
C4	SW	PC	DB	C15	SR	PC	DB
C5	SW	PC	1.5 × DB	C16	SR	PC	1.5 × DB
C6	SW	PC	2.0 × DB	C17	SR	PC	2.0 × DB
C7	SW	PC	3.0 × DB	C18	SR	PC	3.0 × DB
C8	SW	PMA	DB	C19	SR	PMA	DB
C9	SW	PMA	1.5 × DB	C20	SR	PMA	1.5 × DB
C10	SW	PMA	2.0 × DB	C21	SR	PMA	2.0 × DB
C11	SW	PMA	3.0 × DB	C22	SR	PMA	3.0 × DB

Explanations: SW–anthropogenic land soil; SR–reference soil; DB–basic fertiliser dose according to Table 10; PMA–ash from poultry manure incineration; PC–separated phosphate concentrate; CF-S–commercial fertiliser Superfosfat; CF-A–commercial fertiliser Azofoska.

**Table 12:** Substrate preparation for pot cultivation.

Test Symbol	Substrate			Test Symbol	Substrate		
	Soil	Fertiliser			Soil	Fertiliser	
		Type	Dose			Type	Dose
P1	SW	-	-	P8	SW	PC	3.0 × DB
P2	SW	CF-S	DB	P9	SW	PMA	0.5 × DB
P3	SW	CF-A	DB	P10	SW	PMA	1.0 × DB
P4	SW	PC	0.5 × DB	P11	SW	PMA	1.5 × DB
P5	SW	PC	1.0 × DB	P12	SW	PMA	2.0 × DB
P6	SW	PC	1.5 × DB	P13	SW	PMA	3.0 × DB
P7	SW	PC	2.0 × DB				

Explanations: SW–anthropogenic land soil; DB–basic fertiliser dose according to Table 10; PC–separated phosphate concentrate; PMA–ash from poultry manure incineration; CF-S–commercial fertiliser Superfosfat; CF-A–commercial fertiliser Azofoska.

**Table 13:** Fertiliser dosing in plots.

Plot Symbol	Fertiliser		Plot Symbol	Fertiliser	
	Type	Dose		Type	Dose
F1	-	-	F6	PC	2.0 × DB
F2	CF-A	DB	F7	PC	3.0 × DB
F3	CF-S	DB	F8	PMA	0.5 × DB
F4	PC	0.5 × DB	F9	PMA	DB
F5	PC	DB	F10	PMA	2.0 × DB

Explanations: DB–basic fertiliser dose according to Table 10; PC–separated phosphate concentrate; PMA–ash from poultry manure incineration; CF-S–commercial fertiliser Superfosfat, CF-A–commercial fertiliser Azofoska.

## Data Availability

Not applicable.
